# A case of endoscopic selective muscular dissection for calcifying fibrous tumor

**DOI:** 10.1055/a-2411-7427

**Published:** 2024-09-19

**Authors:** Rie Tsukida, Yoichi Yamamoto, Hiroki Sakamoto, Junya Sato, Nobuyuki Oike, Tadakazu Shimoda, Hiroyuki Ono

**Affiliations:** 1 38471 Division of Endoscopy, Shizuoka Cancer Center, Shizuoka, Japan; 2 38471 Division of Diagnostic Pathology, Shizuoka Cancer Center, Shizuoka, Japan

A 67-year-old woman diagnosed with a gastric submucosal tumor (SMT) by esophagogastroduodenoscopy (EGD) during a medical checkup was referred to our hospital. EGD revealed a 20-mm SMT on the lesser curvature of the lower gastric body ( Fig. 1 ). Endoscopic ultrasound (EUS) showed a low-echoic homogeneous lesion, which had echogenic spots with an acoustic shadow and may have been localized within the layer of the muscularis mucosa to the deep submucosa ( Fig. 2 ). EUS fine-needle biopsy was performed, and a strongly suspected calcifying fibrous tumor (CFT) was detected on pathological examination; however, a definitive diagnosis could not be made. Endoscopic selective muscular dissection with en bloc resection was performed as diagnostic treatment. The SMT was diagnosed as CFT on pathological examination ( Fig. 3 ). The vertical margin was negative in the area where the muscle layer was attached, whereas it was inconclusive where the muscle layer was not attached ( Fig. 4 , Video 1 ).

**Fig. 1:**
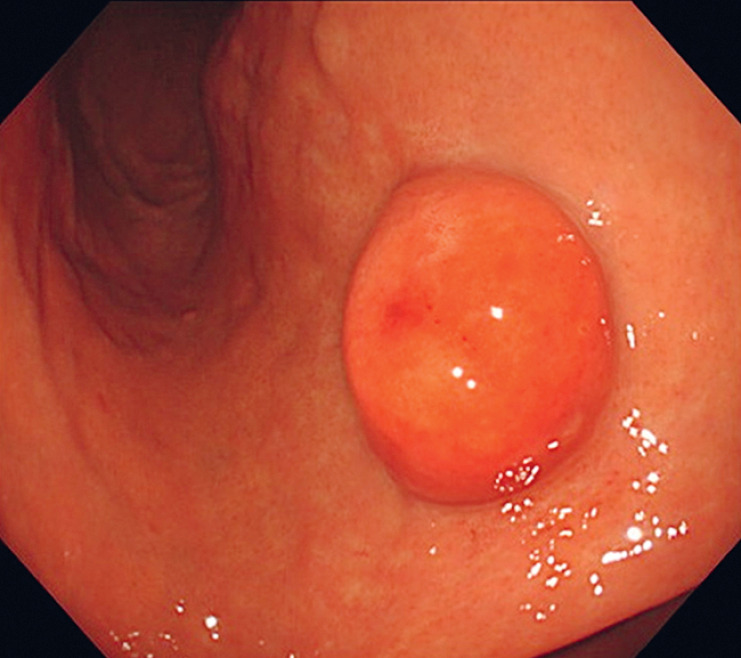
Endoscopic images. On the lesser curvature of the lower gastric body, there is a 20-mm elevated lesion covered by nonneoplastic mucosa.

**Fig. 2:**
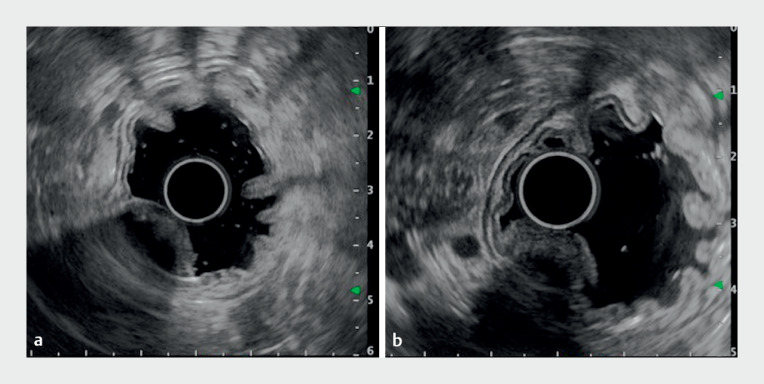
EUS images. **a, b** A 16 × 9-mm low-echoic homogeneous lesion is shown growing into the gastric lumen, with echogenic spots with the acoustic shadow. Due to the deep attenuation, it was obscured, indicating that it may be localized within the layer of muscularis mucosa to deep submucosa.

**Fig. 3:**
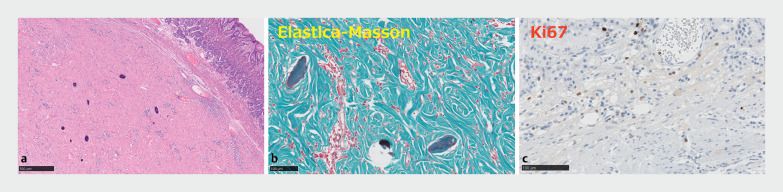
Pathological findings. **a** The tumor consisted of spindle-cells with no atypia and a collagenous matrix, with infiltration of inflammatory cells, mainly lymphocytes and plasma cells, and some psammoma bodies. **b** There is a great increase in the number of mature collagen fibers. **c** Immunohistochemical results showed a very low Ki67 labeling index (less than 1.0%).

**Fig. 4:**
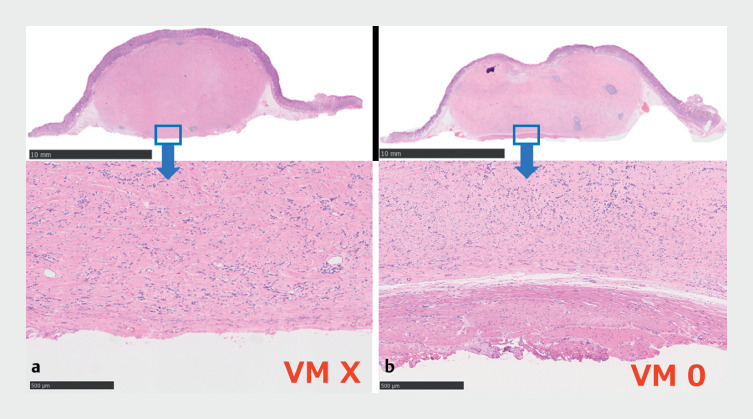
**a** The vertical margin was inconclusive in the area where the muscle layer was not attached. **b** The vertical margin was negative in the area where the muscle layer was attached.

Endoscopic selective muscular dissection for calcifying fibrous tumor.Video 1

Gastric CFTs are difficult to diagnose before treatment 1 . Although gastric CFTs are typically treated surgically, endoscopic resection has been reported in some recent cases 2 3 . Gastric CFTs can originate from all layers, but most commonly the submucosa 1 . Endoscopic submucosal dissection (ESD) may be an inadequate treatment for CFTs of the deep submucosa. Endoscopic selective muscular dissection has been reported as a new treatment strategy for cT1b gastric cancer 4 . Endoscopic selective muscular dissection is the method of dissection between the oblique and circular muscle layer, not the submucosa. In this case, the vertical margin was negative in the area where the muscle layer was attached, whereas it was inconclusive in the area where muscle layer was not attached. In endoscopic selective muscular dissection, VM0 resection may be possible if we attempt to dissect the muscular layer in the wider area. Endoscopic selective muscular dissection may be a useful treatment option for patients with CFT suspicious of being located in the deep submucosa.

Endoscopy_UCTN_Code_CCL_1AB_2AD_3AB

## Notes

Endoscopy E-Videos https://eref.thieme.de/e-videos
*E-Videos* is an open access online section of the journal *Endoscopy* , reporting on interesting cases and new techniques in gastroenterological endoscopy. All papers include a high-quality video and are published with a Creative Commons CC-BY license. Endoscopy E-Videos qualify for HINARI discounts and waivers and eligibility is automatically checked during the submission process. We grant 100% waivers to articles whose corresponding authors are based in Group A countries and 50% waivers to those who are based in Group B countries as classified by Research4Life (see: https://www.research4life.org/access/eligibility/ ). This section has its own submission website at https://mc.manuscriptcentral.com/e-videos .
